# Two conformational states in D-shaped DNA: Effects of local denaturation

**DOI:** 10.1038/srep28239

**Published:** 2016-06-24

**Authors:** O.-chul Lee, Cheolhee Kim, Jae-Yeol Kim, Nam Ki Lee, Wokyung Sung

**Affiliations:** 1 Department of Physics and Postech Center for Theoretical Physics, Pohang University of Science and Technology, Pohang 790-784, Republic of Korea; 2 Center for Self-assembly and Complexity, Institute for Basic Science (IBS), Pohang 37673, Republic of Korea; 3 Department of Physics, Pohang University of Science and Technology, Pohang 790-784, Republic of Korea

## Abstract

The bending of double-stranded(ds) DNA on the nano-meter scale plays a key role in many cellular processes such as nucleosome packing, transcription-control, and viral-genome packing. In our recent study, a nanometer-sized dsDNA bent into a D shape was formed by hybridizing a circular single-stranded(ss) DNA and a complementary linear ssDNA. Our fluorescence resonance energy transfer (FRET) measurement of D-DNA revealed two types of conformational states: a less-bent state and a kinked state, which can transform into each other. To understand the origin of the two deformed states of D-DNA, here we study the presence of open base-pairs in the ds portion by using the breathing-DNA model to simulate the system. We provide strong evidence that the two states are due to the emergence of local denaturation, i.e., a bubble in the middle and two forks at ends of the dsDNA portion. We also study the system analytically and find that the free-energy landscape is bistable with two minima representative of the two states. The kink and fork sizes estimated by the analytical calculation are also in excellent agreement with the results of the simulation. Thus, this combined experimental-simulation-analytical study corroborates that highly bent D-DNA reduces bending stress via local denaturation.

DNA is an important biological molecule that carries the complete genetic information of an organism. On the submicron scale, DNA assumes a variety of conformations for various biological functions^1^. Although double-stranded (ds) DNA has a persistence length (*L*_*ds*_) of approximately 50 nm^2^, it has been shown to bend sharply on the nanoscale (i.e., much shorter than *L*_*ds*_) in many biological processes such as gene regulation^3^, transcription factor binding^4^,^5^ and DNA packaging^6^. The possibility of sharp bending has been shown by cyclization experiments of DNA. Remarkably, the cyclization rates of short DNA are found to be considerably greater than those expected based on the predictions of the worm-like-chain (WLC) model^7^ which treats DNA as an elastic rod^8^,^9^. Recently, Vafabakhsh and Ha used a FRET-based single-molecule assays to measure the looping dynamics of short dsDNA in the absence of proteins^10^. The cyclization factors were measured to be several orders of magnitude greater than those predicted by the WLC model for DNA lengths below 100 base pairs (bp). This phenomenon implied an increase in dsDNA flexibility, possibly due to a flexible hinge in the form of kinks and bubbles that can form under strong bending^11^,^12^,^13^,^14^,^15^.

In a recent study, Qu *et al*.^16^,^17^ designed short D-shaped DNA by hybridizing two ssDNA, where D-shaped DNA contains a dsDNA portion with a nick in the middle and ssDNA portions of various lengths. By using gel electrophoresis in equilibrium, they showed the transition and coexistence between two conformational states: a less-bent state and a sharply kinked state, where the length of the ds and ss portions are 18 bp and 26 bp, respectively^16^. In a more recent study, for D-shaped DNA without nick formed by hybridizing an ss loop with a linear complementary ss, they showed that the ds portion transforms from a less-bent state to sharply kinked state when the bending torque on the ds exceeds a certain value^17^. However, their studies, based on electrophoresis gel experiments for bulk DNA, are quite limited with regard to detailing the conformations and conditions for DNA transition and coexistence.

Recently, we performed single-molecule experiments in which we applied alternating laser excitation fluorescence resonance energy transfer (ALEX-FRET)^19^,^20^ to D-shaped DNA composed of 30 bp ds portions and ss portions of various lengths^18^. The FRET signal from the two dyes attached to the ds portion near both ends (see Fig. 1) indicated that as the ss portion shortened below 16 bp, the ds portion bifurcated to short- and long-end-to-end distance (EED) states. The emergence of the two states in the ds portion differs from that detected by an earlier experiment of Qu *et al*. performed on nicked DNA, suggesting that this emergence is due to the large bending stress applied to the ds portion. The two states can dynamically interconvert between each other in milliseconds. Furthermore, the experiment^18^ showed that D-shaped DNA with a 3-bp size mismatch in the center of the ds portion yields a FRET signal indistinguishable from that of the short-EED state. This result suggested that local melting was responsible for the short-EED state, which arises to release the large bending stress.

Motivated by this experiment, we now turn to numerical simulations and analytical modeling to understand the mechanism that leads to the emergence of the two states in highly stressed DNA. We consider D-shaped DNA with a 30-bp-long ds portion and a 10-bp-long ss portion in a buffer solution with 10 mM Mg^2+^ concentration, where the two states distinctly coexist. We base the simulation on the breathing-DNA model^21^,^22^,^23^ to obtain information on the distance between two beads whose positions are set to be the same as those of the FRET-labeled bases in the ds portion. We find that the EED distribution is consistent with the distribution deduced from the FRET experiment, particularly in that the distributions have two peaks, which correspond to two minima in the associated free energy. To clarify the origin of the bistability, we further investigate the probability of each bp opening in ds portion at each region of the free energy minima. The simulation result indicates that the DNA has a kinked state because of a bubble nucleated in the middle of the ds portion and a less-bent state with a pair of forks at both ends. Finally, we develop an analytical model that indicates that the bistability arises via the formation of a kink of about 5-bp length and two forks of about 3-bp length in order to release the large bending stress.

## Results

### FRET experiment of D-shaped DNA

**Figure 1:**
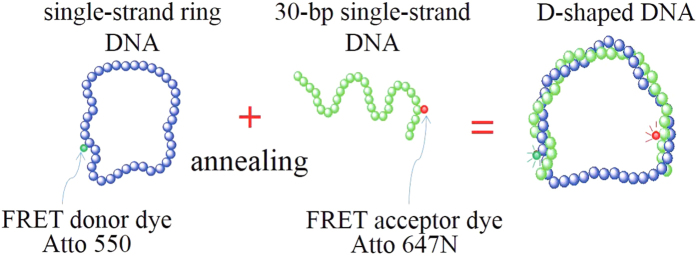
Schematic of D-shaped DNA formation. D-shaped DNA is designed by hybridizing two ssDNA, which consist of one ring ss of varying length and a 30-bp-long linear ss. The 30-bp-long ds portion is labeled with donor (Atto 550) and acceptor (Atto 647 N) fluorophores. The sequence of ds portion in the ring ss is CCTTAGAACAGATCGCACCTATTGATATGG. The linear ss has the 30 nt complementary sequences, GGAATCTTGTCTAGCGTGGATAACTATACC.

We measured the FRET distributions of D-shaped DNA formed by hybridizing circular ssDNA (contour length *Ns* = 34–46 bp) with a linear ssDNA of length *N*_*l*_ = 30 bp (ref. 18 for the experimental details). The ds portion is bent in an arc, whereas the remaining ss portion is linearly stretched. The two positions located 4 bp inward from each end of the arc labeled with donor and acceptor fluorophores, as shown in Fig. 1. The FRET efficiency (*E*) is sensitive to changes in the distance *R* between donor and acceptor and thus provides information regarding this distance.

**Figure 2:**
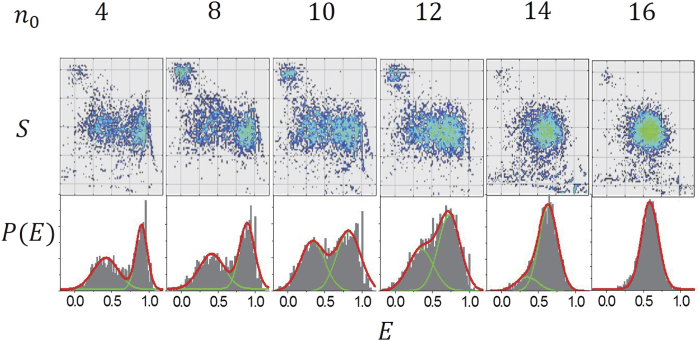
Results of single-molecule FRET experiment with D-shaped DNA for ss length (*n*_0_) varying from 4 bp to 16 bp in a buffer solution with 10 mM Mg^2+^ concentration. Upper row shows two-dimensional histograms of the stoichiometry *S* vs FRET efficiency *E (S* = 1 is donor-only results and *S* = 0 is acceptor-only results). Lower row shows the FRET-efficiency density of D-shaped DNA vs FRET efficiency *E*.

**Figure 3:**
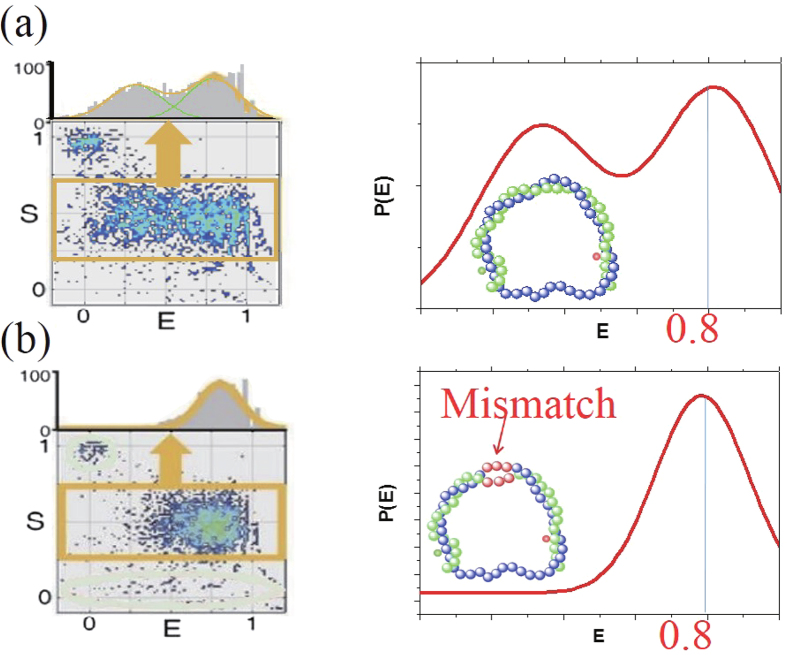
Intensity of FRET efficiency *E* in D-shaped DNA (a) without and (b) with a mismatch in the middle of the arc portion for an ss length (*n*_0_) of 10 bp. The high *E* for the peaks are identical; *E* = 0.8. This FRET data show that the mismatch shortens the EED in the same manner as the presumed kink does.

Unlike the previous experiment^18^ which used upto 5 mM Mg^2+^ concentration, in this work we consider 10 mM Mg^2+^ concentration, which is relevant to the biological condition in cells. Our new experiment results are shown in Fig. 2. Each dot in the upper part of Fig. 2 represents the value of *E* for each single D-shaped DNA in a buffer solution with 10 mM Mg^2+^ concentration, where *n*_0_ = *N*_*s*_ − *N*_*l*_ is the length of the ssDNA string and ranges from 4 bp to 16 bp (see Fig. 1). From the data, which are represented by dots, the intensity *P*(*E*) of a given *E* is obtained as shown in the lower part of Fig. 2. As the ss length *n*_0_ increases from 4 bp to 16 bp, the structure of the peaks in *P*(*E*) changes; *P*(*E*) has two peaks for *n*_0_ = 4–14 bp, whereas above 15 bp, only a single peak appears. In particular, for *n*_0_ = 10 bp, *P*(*E*) has two peaks of nearly equal height at different FRET efficiencies. The two corresponding states are depicted in detail in Fig. 3(a). The two peaks have been attributed to the two conformational states of D-shaped DNA: a kinked state with short EED (high *E*) and a less-bent state with long EED (low *E*)^18^ Inserting a 3-bp mismatch in the middle of the arc, as shown in Fig. 3(b), for *n*_0_ = 10 bp, gives a FRET efficiency *E* = 0.8. Again, this value is indistinguishable from the large *E* value for the fully matched DNA^18^, thus corroborating the emergence of the kinked state.

### Breathing-DNA model

We use the breathing-DNA model to understand in detail the physical mechanism underlying the conformational change in short dsDNA due to bending force. This model is used to study how bp-bond fluctuations affect short-DNA flexibility, especially in strongly bent situations. The model describes the emergence of bp openings, which are called bubbles and are induced by thermal fluctuations^21^,^23^ and external bending^22^. In previous work, we applied this model to short DNA and found that the effective persistence length of dsDNA decreases as its length decreases because of the presence of bubbles and forks^21^ and that, to release mechanical stress, bubble formation and growth is facilitated by bending^22^. From these studies, we gained confidence that the breathing-DNA model reasonably describes dsDNA phenomena involving nanoscale denaturation behaviors.

**Figure 4:**
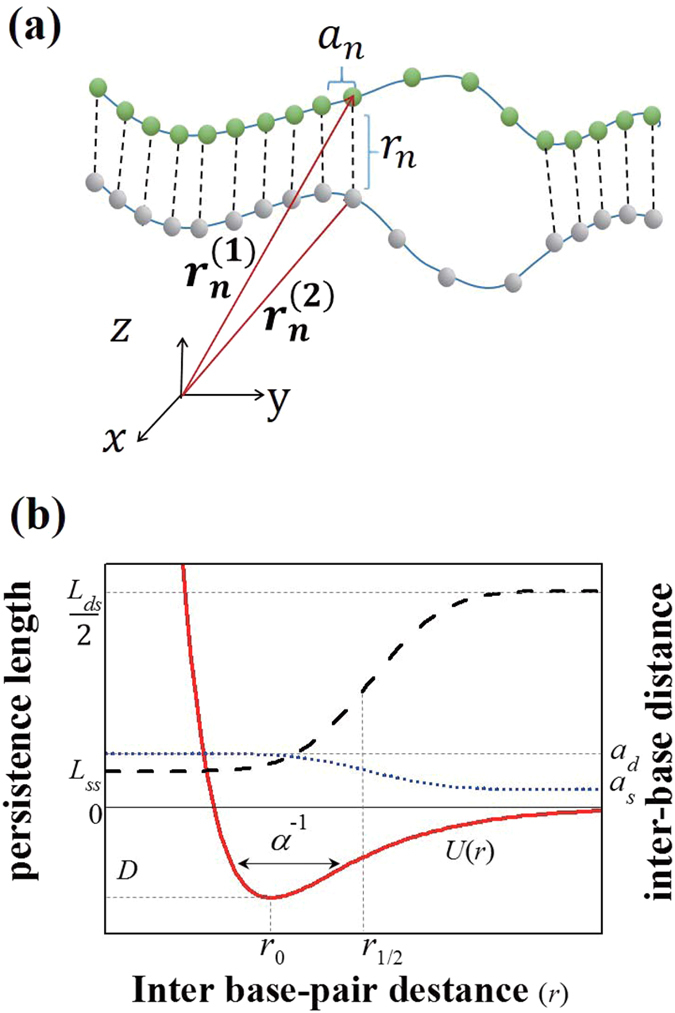
(**a**) Schematic figure of breathing-DNA model. Here, 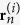 and *r*_*n*_ are three-dimensional position vectors of the *n* th bead in the *i*th strand and bp distance between *n*th beads, respectively. The quantity *a*_*n*_ is the inter-base distance in a single strand. (**b**) Schematic figure showing the profile of persistence length *L*_*p*_ (dashed line) and inter-base distance *a*_*n*_ (dotted line) per single strand along with the Morse potential *U*(*r*) (solid line) as a function of bp distance *r*.

In the breathing-DNA model, we consider the effective Hamiltonian


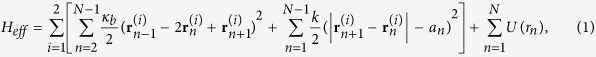


where 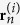 is a three-dimensional position vector of the *n*th bead in the *i*th strand, as shown in Fig. 4(a). The first term gives the bending energy of the duplex, where *κ*_*b*_ is the bending modulus of each strand. The second term gives the stretching energy, which is introduced to impose the chain-inextensibility condition. In this term, *k* and *a*_*n*_ are the stretching modulus and inter-base distance for each chain, respectively. The last term is the pairing energy between complementary bases, which we adopt as the Morse potential to describe bp opening due to thermal excitation. The Morse potential is given by


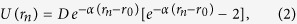


where *r*_*n*_ is the *n*th bp distance and *D* is the potential depth, which we adopt as average of the A-T- and G-C-pair binding energies. The quantities *r*_*0*_ and *α*^−1^ are the bp mean distance and range characteristic of the mean and fluctuation of the DNA diameter, respectively.

The bending modulus *k*_*b*_ is proportional to the persistence length *L*_*p*_, *k*_*b*_ = *k*_*B*_*TL*_*p*_. As shown by the dashed line in Fig. 4(b), when three consecutive bps are separated by more than *r*_1/2_ (i.e., they are in open state), *L*_*p*_ takes on the ss persistence length (*L*_*ss*_ = 3 nm). However, if any one of the bps are bound, *L*_*p*_ is reduced to half the ds persistence length (*L*_*p*_ = *L*_*ds*_/2 =25 nm)^21^,^22^. The *a*_*n*_ is the inter-base distance which is assumed to vary as a function of bp distances from the ds value *a*_*d*_ to the ss value *a*_*s*_, as follows:


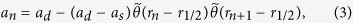


where 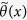 is the step-like function


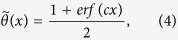


which smoothly increases from 0 to 1 with a characteristic length *c*^−1^ ~ α^−1^. When two consecutive bps are opened, *a*_*n*_ takes on the ss inter-base distance of 0.7 nm. In contrast, when either of the two bps are bound, *a*_*n*_ becomes the distance between nearest-neighbor bps in the ds, as shown by the dotted line in Fig. 4(b). For our model, the parameter in the Morse potential *U*(*r*) is chosen for a long DNA to denature at a melting temperature (350 K). The resulting values are *D*  = 0.07 eV, *α* = 20 nm^−1^, and *c* = 21.3 nm^−1 ^22,23. We use *k *= 0.45 eV ps/nm^2^ to fit the force-extension curve for the 300 bp-free DNA fragments^21^. In this model, varying persistence length and bead–bead distance describes the stacking interactions that underlie the helical structure.

By applying this energy model, we simulate the Langevin dynamics for each bead (representative of each base):


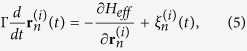


where Γ = 1.88 × 10^−11^ kg/s is the frictional coefficient per bead^21^,^22^,^23^, 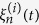 is the Gaussian and white noise, which are characterized by 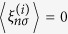 and 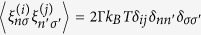, for each Cartesian component *σ* and *σ*′.

**Figure 5:**
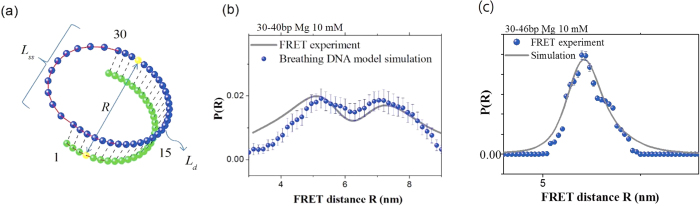
(**a**) Initial configuration of simulation. The distance *R* between the yellow and red beads is set to the distance between bases, which are labeled with FRET dye in the experiment. (**b**) Probability density of FRET distance for *n*_*0*_ = 10 bp. The solid line represents the FRET-experiment results, whereas the filled circles represent the results from the breathing-DNA simulation. (**c**) Probability density of FRET distance for *n*_*0*_ = 16 bp. The error bars of simulation results are obtained from 50000 times repeated simulations.

The simulation was performed by forming a D-shaped DNA configuration with a 30-bp-long ds portion and a 10-bp-long ss portion at room temperature (295 K) in conformity with the experiment [see Fig. 5(a)]. The simulations is done for 10^7^ time-steps (10^−3^ s) where one time step Δ*t* is 0.1 ns. In the simulation, the initial state is chosen to be the D-DNA conformation with all bps completely bound as shown in Fig. 5(a). As times goes on, the local melting begins to occur due to thermal fluctuation. After the system is relaxed to equilibrium in the time longer than 1 *μs*, the data for the bp positions are collected to extract the information of EED (FRET distance) *R* between the labeled beads. Figure 5(b,c) show the probability distribution *P*(*R*) for *n*_*0*_ = 10 bp and *n*_*0*_ = 20 bp. First, from the experimental results, we need to convert the FRET efficiency *E* in the experiment to the FRET distance *R* via the equation


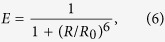


where *R*_0_ = 6.5 nm is the Förster distance of this donor-acceptor pair (i.e., the distance at which the energy-transfer efficiency is 50%). The probability distribution *P*(*R*) is given by *P*(*R*) = *P*(*E*)*dE*/*dR*, which is plotted as the solid line in Fig. 5(b,c). Although we ignored the linker length and the dye size in this simulation, a reasonable agreement is obtained between the simulation results (filled dots) and experimental results (solid line). In particular, the two distances *R* at which *P*(*R*) peaks, approximately 5.2 nm and 7.0 nm, are nearly the same. The discrepancy between the simulation and experimental results are due to the factors that contribute to the broadness of the FRET distributions. For example, the fluctuation of linker lengths of fluorescence dyes, rotational distributions, and the shot noise. These fluctuations may increase the population of low and high FRET regions, compared with the simulations. As a result, the simulation presents smaller population for the long and short *R* values.

**Figure 6:**
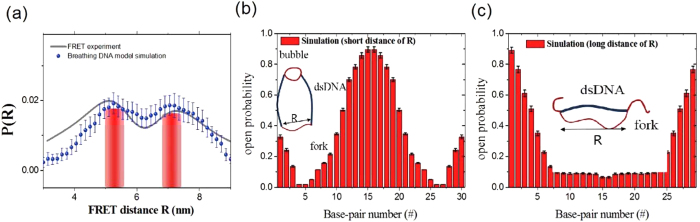
(**a**) Probability density associated with EED *R* in D-shaped DNA. The bars represent the two-state region: a kinked state (5.1–5.4 nm) and a less-bent state (6.8–7.2 nm). Figure 5(b) is redrawn to highlight the two state regions. (**b**) The open probability vs segment number in the ds portion when the distance *R* is in the range 5.1–5.4 nm. (**c**) The open probability vs segment number in the ds portion when the distance *R* is in the range 6.8–7.2 nm.

To understand the physical mechanism that gives rise to the two peaks, we study the probability of each bp opening along the ds portion when the distance *R* is in the ranges 5.1–5.4 nm and 6.8–7.2 nm, as depicted by the red regions, representative of two probability maxima in Fig. 6(a). Figure 6(b,c) depict the opening probability of each bp in the ds portion for two different states. In the conformal state where DNA has a relatively short distance *R*, the bp opening probability is concentrated in the middle of the ds portion. This result means that *R* for the short EED is induced by forming a nucleated bubble with an average size 5.3 bp [marked by a loop in a schematic diagram in Fig. 6(b)]. In sharp contrast, for a relatively large distance *R*, the opening probability is depleted from the middle but is concentrated near the end of the arc [see Fig. 6(c)]. This form of bp opening is called a fork. Figure 6(c) depicts the conformation expected with the ds portion (bold line) and two forks (tails at both ends). By forming the forks, the ds arc can reduce the free energy of the bending ds. From the simulation, we obtain an average bubble size and fork size of 5.3 bp and 3.3 bp, respectively.

## Analytical model

To understand the free-energy bistability for D-shaped DNA along with the emergence of a kink and of forks with the sizes mentioned above, we study an analytical model. We consider the total free energy as the sum of the bending energy *F*_*bend*_ from the ds portion bent into a D shape, the denaturation energy *F*_*den*_ from a bubble and two forks, and the stretching energy *F*_*st*_ on the remaining ss portion:


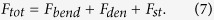


Assuming that the centerlines of the duplex and bubble form two arcs with different bending angles *θ*_*s*_ and *θ*_*d*_ per base, respectively, and the whole configuration has right-left symmetry, the bending energy is


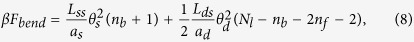


where *n*_*b*_ and *n*_*f*_ are the bp length of the bubble and the forks and *β* = 1/*k_B_T*. Second, the free energy associated with the denaturation is given by


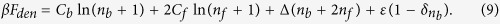


Here, the first two terms represent the entropy cost of forming a bubble and two forks, with the statistical factors *C*_*b*_ = 1.7 and *C*_*f*_ = 1.1^21^,^23^. The last two terms represent the bp unbinding energy and bubble-initiation energy, where Δ = 0.33 *k*_*B*_*T* and *ε* = 5 *k*_*B*_*T*^21^,^22^. Finally, for the ss stretched by a putative force *f*_*s*_, we use the freely jointed-chain model^24^ which gives the EED *z* as


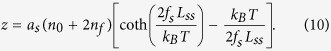


**Figure 7:**
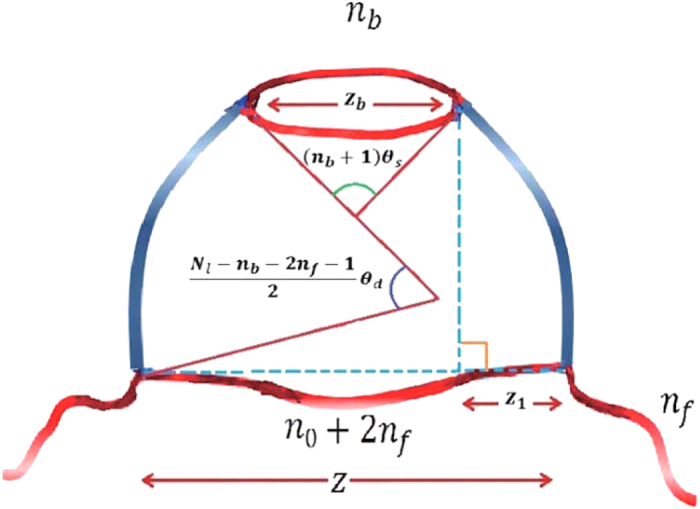
Schematic for theoretical model. Two ds portions and a bubble (length *n*_*b*_) form circular arcs, with different bending angle per bp. The string is formed from an ss portion (*n*_0_= 10 bp) and two forks (each of length *n*_*f*_). The high bending force on the ds is balanced by the stretching force on the ss.

By using Eq. 10, we obtain the stretching free energy 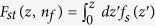 with *n*_0_ = 10 bp as a function of *z* and *n*_*f*_. However, from the geometry of the conformation, *z* is given by (see Fig. 7)


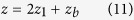


where


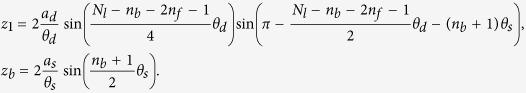


From Eq. 11, the stretching free energy *F*_*st*_(*z, n*_*f*_) is given as a function of *F*_*st*_(*n*_*b*_, *n*_*f*_, *θ*_*s*_, *θ*_*d*_). By using the condition of balance *f*_*s*_ = *f*_*d*_, where *f*_*b*_ is the force to bend the ds portion:


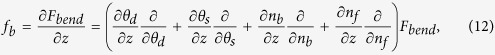


so we can eliminate the variable *θ*_*d*_ to express the total free energy as a function of the remaining three variables *n*_*b*_, *n*_*f*_ and *θ*_*s*_ (*F*_*tot*_(*θ*_*s*_, *n*_*b*_, *n*_*f*_)).

**Figure 8:**
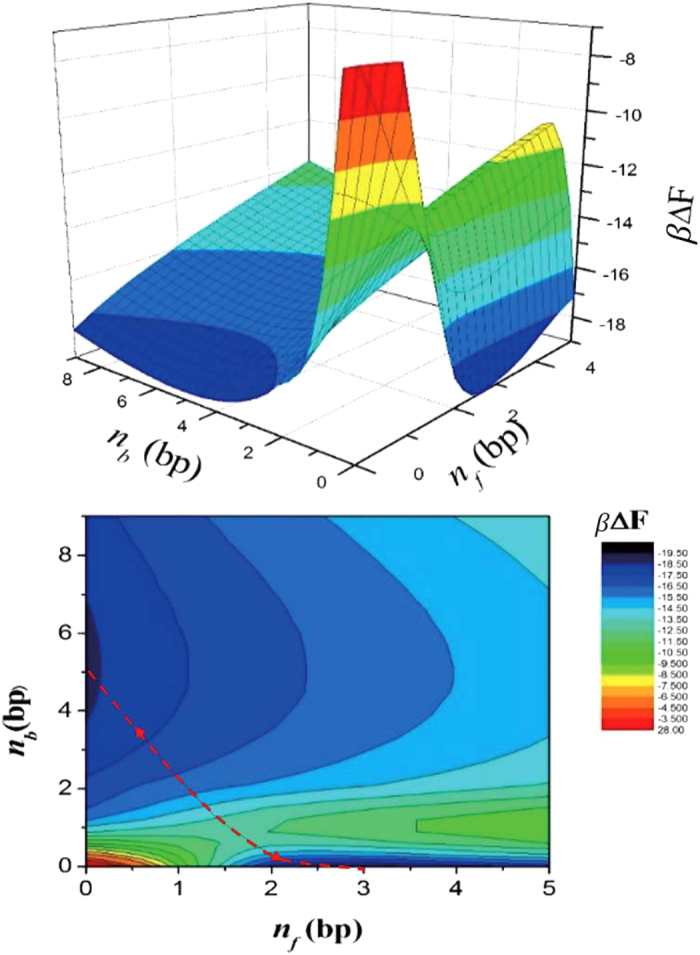
*β*ΔF(*n*_*b*,_ *n*_*f*_), the difference between the total free energy and the contribution from the ds portion free of bubble and forks, as a function of bubble and fork size. This plot shows two minima in the free energy: one at *n*_*b*_ = 5 and *n*_*f*_ = 0 and another at *n*_*b*_ = 0 and *n*_*f*_ = 3. The dashed line in the lower graph is pathway for the transition along valley of the free energy landscape.

Finally, keeping the two variables *n*_*b*_ and *n*_*f*_ fixed, the total free energy is minimized by varying *θ*_*s*_. Figure 8 shows the minimized free energy, which is now plotted as a function of *n*_*b*_ and *n*_*f*_. Here we plot the difference between the total free energy and the contribution from the ds portion free of bubble and forks: Δ*F*(*n*_*b*,_ *n*_*f*_) = *F*_*tot*_(*θ*_*s*_(*n*_*b*_, *n*_*f*_), *n*_*b*,_ *n*_*f*_) − *F*_*tot*_(*θ*_*s*_(0.0), 0, 0). Figure 8 shows that Δ*F*(*n*_*b*_, *n*_*f*_) has two minima: one at *n*_*b*_ = 5 and *n*_*f*_ = 0 and another at *n*_*b*_ = 0 and *n*_*f*_ = 3, which explains the emergence of the bubble and forks. In fact, the sizes of the bubble and forks are consistent with those obtained from the simulation results (bubble size is 5.3 bp and fork size is 3.3 bp), which attests to the internal consistency of our two-state model. For *n*_*b*_ = 5 and *n*_*f*_ = 0 (i.e., the kinked state), we obtain an EED of *z* = 5.692 nm, which is also consistent with the results of the FRET experiment and the breathing-DNA simulation (approximately 5.2 nm). For *n*_*b*_ = 0 and *n*_*f*_ = 3 (i.e., the less-bent state), however, *z* = 8.84 nm, which is about 15% longer than the results of the FRET experiment and the simulation. This discrepancy is attributed to the fact that in this analysis, *z* is literally the EED of the ds portion, whereas, in reality, it is the distance between two beads located 4 bp inward from the ends, as mentioned earlier.

## The transition kinetics

**Figure 9:**
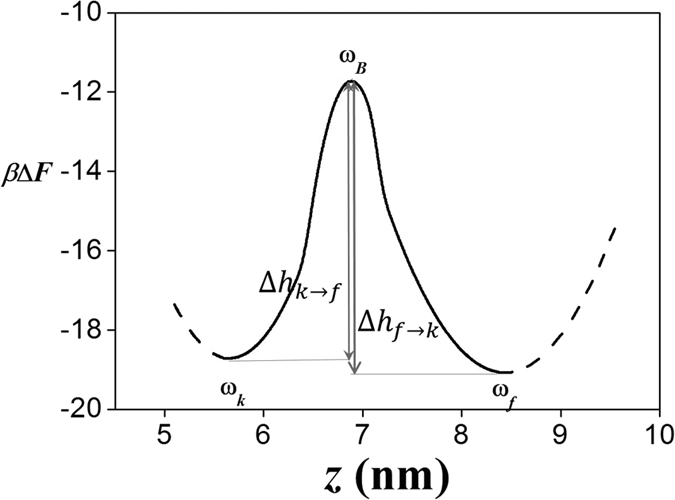
*β*ΔF(*n*_*b*_, *n*_*f*_) represented as a function of EED, using *z*(*n*_*b*_, *n*_*f*_). *z* is calculated for the reaction coordinates (*n*_*b*_, *n*_*f*_) on the dominant pathway (dashed line in Fig. 8).

The dominant pathway of the transition is along the valley in the two-dimensional free-energy landscape, the locus of the lowest free energy. Figure 9 shows the free-energy landscape when the reaction coordinates (*n*_*b*_, *n*_*f*_) move along the valley from one well to the other. The free energy is drawn as the function of EED, *z* = *z*(*n*_*b*_, *n*_*f*_), which can be regarded as a one-dimensional coordinate for the reaction. A relatively high energy barrier Δ*h* ~ 7 *k*_*B*_*T* is apparent at *n*_*b*_ = 1 in the two-dimensional landscape (Fig. 8), which is attributed to the energy cost of bubble initiation. The rate of crossing the free-energy barrier is given by Kramer’s theory:


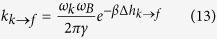



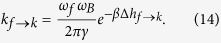


Here, *ω* is the curvature parameter 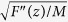 where *M* ~ 4 × 10^−23^ kg is total mass of D-DNA: at the kinked state, 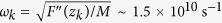 and at the fork state, 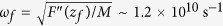, whereas at the barrier height, 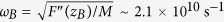 . The coefficient *γ* is the effective friction coefficient associated with the transitions, which are regarded to be same for both ways. Substituting into the formulae the value *k*_*k *→ *f*_ = 719 s^−1^ obtained experimentally, we obtain *γ* = 6.5 × 10^13^ s^−1^. Substituting this result into the formulae, we find *k*_*f *→ *k*_ = 535 s^−1^, which is remarkably consistent with the experimental result (*k*_*f *→ *k*_ = 498 s^−1^). The higher rate *k*_*k *→ *f*_ is attributed to the fact that *ω*_*k*_ > *ω*_*f*_. Physically, this is explained by the wide fluctuation in EED for the fork state.

## Summary

With a single-molecule experiment, we detect that a short dsDNA strongly bent into a D-shape has two conformational states. One state is the ds short-EED state caused by a kink, as suggested by the same experiment with a mismatch placed in the middle of the dsDNA contour. To understand the mechanism that causes the two states to emerge, we perform a Brownian dynamic simulation based on the breathing-DNA model. This simulation indicates that one state is indeed induced by a kink or a bubble nucleated from the unbound base pairs, whereas the other state with longer EED is caused by local melting resulting in forks forming at the ends of the dsDNA.

By using an analytical model, we obtain the bubble size *n*_*b*_, the fork size *n*_*f*_, and the free-energy landscape as a function of these two variables. With these results, we find two minima: one at (*n*_*b*_, *n*_*f*_) = (5, 0), and another at (*n*_*b*_, *n*_*f*_) = (0, 3). The first minimum represents the kink state and the second minimum represents the less-bent state; these results are consistent with those obtained from the simulation.

This results indicate that under a high bending stress, dsDNA locally melts to release the stress. This research thus suggests that small-size DNA looping can be facilitated by local melting, in particular, by the formation of a nucleated bubble, namely the kink.

## Additional Information

**How to cite this article**: Lee, O.-chull. *et al*. Two conformational states in D-shaped DNA: Effects of local denaturation. *Sci. Rep.* **6**, 28239; doi: 10.1038/srep28239 (2016).
